# An explainable unsupervised learning approach for anomaly detection on corneal *in vivo* confocal microscopy images

**DOI:** 10.3389/fbioe.2025.1576513

**Published:** 2025-06-06

**Authors:** Ningning Tang, Qi Chen, Yunyu Meng, Daizai Lei, Li Jiang, Yikun Qin, Xiaojia Huang, Fen Tang, Shanshan Huang, Qianqian Lan, Qi Chen, Lijie Huang, Rushi Lan, Xipeng Pan, Huadeng Wang, Fan Xu, Wenjing He

**Affiliations:** ^1^ Guangxi Key Laboratory of Eye Health and Guangxi Health Commission Key Laboratory of Ophthalmology and Related Systemic Diseases Artificial Intelligence Screening Technology and Research Center of Ophthalmology, Guangxi Academy of Medical Sciences and Department of Ophthalmology, The People’s Hospital of Guangxi Zhuang Autonomous Region, Nanning, China; ^2^ Information and Technology Department, Guangxi Beibu Gulf Bank Co., Ltd., Nanning, China; ^3^ Guangxi Key Laboratory of Image and Graphic Intelligent Processing, School of Computer Science and Information Security, Guilin University of Electronic Technology, Guilin, China

**Keywords:** corneal disease screening, *in vivo* confocal microscopy (IVCM), unsupervised anomaly detection, deep learning (DL), explainable artificial intelligence

## Abstract

**Background:**

*In vivo* confocal microscopy (IVCM) is a crucial imaging modality for assessing corneal diseases, yet distinguishing pathological features from normal variations remains challenging due to the complex multi-layered corneal structure. Existing anomaly detection methods often struggle to generalize across diverse disease manifestations. To address these limitations, we propose a Transformer-based unsupervised anomaly detection method for IVCM images, capable of identifying corneal abnormalities without prior knowledge of specific disease features.

**Methods:**

Our method consists of three submodules: an EfficientNet network, a Multi-Scale Feature Fusion Network, and a Transformer Network. A total of 7,063 IVCM images (95 eyes) were included for analysis. The model was trained exclusively on normal IVCM images to capture and differentiate structural variations across four distinct corneal layers: epithelium, sub-basal nerve plexus, stroma, and endothelium. During inference, anomaly scores were computed to distinguish pathological from normal images. The model’s performance was evaluated on both internal and external datasets, and comparative analyses were conducted against existing anomaly detection methods, including generative adversarial networks (AnoGAN), generate to detect anomaly model (G2D), and discriminatively trained reconstruction anomaly embedding model (DRAEM). Additionally, explainable anomaly maps were generated to enhance the interpretability of model decisions.

**Results:**

The proposed method achieved an the areas under the receiver operating characteristic curve of 0.933 on internal validation and 0.917 on an external test dataset, outperforming AnoGAN, G2D, and DRAEM in both accuracy and generalizability. The model effectively distinguished normal and pathological images, demonstrating statistically significant differences in anomaly scores (p < 0.001). Furthermore, visualization results indicated that the detected anomalous regions corresponded to morphological deviations, highlighting potential imaging biomarkers for corneal diseases.

**Conclusion:**

This study presents an efficient and interpretable unsupervised anomaly detection model for IVCM images, effectively identifying corneal abnormalities without requiring labeled pathological samples. The proposed method enhances screening efficiency, reduces annotation costs, and holds great potential for scalable intelligent diagnosis of corneal diseases.

## 1 Introduction

Corneal diseases are a leading cause of blindness worldwide, affecting more than 12 million people (Wang et al., 2023; Wang et al., 2014; Gain et al., 2016). These conditions arise from a variety of causes, including infectious and non-infectious keratitis, corneal degeneration, dystrophies, trauma, and other pathologies. If left undiagnosed or untreated, they can lead to significant vision impairment and irreversible blindness. Early detection is critical to reduce the burden of these diseases, highlighting the urgent need for the development of fast and reliable diagnostic methods in clinical practice and public health.

*In vivo* confocal microscopy (IVCM) is a vital noninvasive imaging modality for diagnosing corneal diseases, capable of visualizing cellular components with a resolution of up to 1 micron (μm) (Chiang et al., 2023). For each eye, sequential scanning of the corneal layers generates hundreds of images, creating a substantial amount of data for analysis. While the high-resolution visualization offered by IVCM is invaluable for understanding the corneal microstructure *in vivo*, it also presents challenges: the sheer volume of data demands sophisticated, accurate, and efficient analytical methods. Manual analysis of IVCM images is not only labor-intensive and time-consuming but also requires a high level of expertise and is prone to subjectivity. These limitations underscore the urgent need for efficient and objective approaches to interpret the data. Artificial intelligence (AI) has emerged as a promising tool to address these challenges, offering potential solutions for automating IVCM image analysis and enhancing both accuracy and efficiency.

While AI shows great potential for automating IVCM image analysis, its current applications struggle to address the diverse and complex nature of corneal diseases. Existing models are often constrained to recognizing specific disease types that they have been trained on, rendering them ineffective when faced with novel or untrained pathologies. This limitation significantly undermines their clinical utility, especially in scenarios involving rare or atypical diseases. Moreover, these shortcomings are exacerbated by the reliance on large, annotated datasets for training. The preparation of such datasets is not only labor-intensive and time-consuming but also highly impractical for rare conditions where sufficient data is inherently scarce. These issues stem from the predominant use of supervised learning frameworks (Lv et al., 2020; Xu et al., 2021; Tang et al., 2023; Tang et al., 2023; Xu et al., 2021), which inherently depend on predefined categories and extensive labeled data, limiting their adaptability and scalability in real-world applications.

Given these challenges, there is a critical need for an efficient and scalable approach to analyze the vast volume of IVCM data. Unsupervised anomaly detection presents a promising solution by learning the characteristics of normal images and identifying deviations that indicate potential abnormalities (Bergmann et al., 2021). Unlike supervised methods, this approach relies solely on normal samples for training, thereby reducing the dependence on extensive labeled datasets. More importantly, it enables the detection of a significantly broader spectrum of abnormalities, including rare and previously unseen conditions, effectively minimizing the risk of missed diagnoses. By identifying deviations from normal patterns, it not only addresses the heterogeneity of corneal pathologies but also helps clinicians swiftly focus on key abnormal images within large datasets. This capability simplifies data preparation, enhances diagnostic efficiency, and expands the scope of detectable conditions, making it a highly adaptable and scalable tool for real-world applications in corneal disease diagnosis.

Despite the promising potential of unsupervised anomaly detection, its application in ophthalmic imaging remains limited, with most studies focusing on retinal imaging. For instance, Jebril et al. (2024) utilized a deep learning-based anomaly detection model to identify abnormal retinal perfusion in optical coherence tomography angiography (OCTA). Luo et al. (2024) developed multi-resolution auto-encoder models to detect unknown retinal diseases using OCT and fundus images, while Ezhei et al. (2022) employed a restricted Boltzmann machine to enhance and analyze retinal OCT images for improved diagnosis. Among the few studies addressing corneal imaging, Yousefi et al. (2018) applied an unsupervised clustering algorithm to corneal OCT images, successfully identifying and monitoring keratoconus stages.

However, research on unsupervised anomaly detection in corneal *in vivo* confocal microscopy (IVCM) image analysis remains largely unexplored. Unlike the above images, IVCM images present unique challenges due to the structural heterogeneity of the cornea. Each corneal layer—epithelium, subepithelial nerve plexus, stroma, and endothelium—exhibits distinct normal morphological characteristics (Cañadas et al., 2022). This diversity within normal images complicates the learning process for unsupervised models, which must effectively capture the normal features of multiple classes to identify abnormalities. Addressing these challenges is critical for advancing the accuracy and applicability of anomaly detection in IVCM images, highlighting the need for further research in this domain.

In this study, we propose an innovative unsupervised anomaly detection approach for IVCM images, leveraging transformer-based architectures and image reconstruction techniques. Our method utilizes only normal datasets during training, addressing the challenge of limited annotated data. We hypothesized that this approach could effectively learn the distinctive characteristics of multiple normal corneal layers within a unified framework, enabling the model to detect a broad spectrum of abnormalities across various corneal layers and lesion types. To evaluate the performance of the proposed model, we conducted experiments on datasets containing diverse normal and pathological images and compared its performance with state-of-the-art unsupervised anomaly detection models, including anomaly detection with generative adversarial networks (AnoGAN), generate to detect anomaly model (G2D), and discriminatively trained reconstruction anomaly embedding model (DRAEM). Subsequently, we performed a quantitative analysis of anomaly scores and generated explainable anomaly maps to visualize the anomalous regions identified by the model. We present this article in accordance with the STARD reporting checklist.

## 2 Materials and methods

### 2.1 Data preparation

#### 2.1.1 Data sets

The data for this study were retrospectively collected. Retrospective data, collected between January 2022 and June 2023, were used to construct the training and internal validation sets. Prospective data, collected between January and July 2024, were used to construct the external test set. All data were collected from the Department of Ophthalmology at Guangxi Zhuang Autonomous Region People’s Hospital.

The training set comprised only normal IVCM images, while the validation and test sets included both normal and anomalous images. For the anomaly detection task, the anomalous class, defined as pathological images exhibiting deviations from the normal corneal structure, was designated as the positive class, while normal images were categorized as the negative class. The inclusion criteria for normal samples were as follows: 1) no ocular discomfort or visual symptoms; slit-lamp examination revealed no corneal abnormalities, including negative fluorescein staining and keratic precipitates; 2) no history of corneal disease, eyelid disorders, glaucoma, or ocular inflammation; and 3) no history of ocular trauma or surgery. The inclusion criteria for anomalous samples required a confirmed diagnosis of corneal abnormalities, including infectious or non-infectious keratitis, corneal degeneration, corneal dystrophy, chemical injury, or corneal leukoma. Among them, cases of infectious keratitis were supported by microbiological evidence, including smears and/or cultures from corneal scrapings, as well as metagenomic next-generation sequencing (mNGS) for pathogen detection when clinically indicated. Images with poor quality or unclear features were excluded to ensure clarity and representativeness.

To avoid dataset contamination and artificially inflated evaluation metrics, images were allocated by eye to ensure that data from the same eye did not appear in multiple datasets. For retrospective data, 85% of normal images were allocated to the training set, and 15% to the internal validation set. All pathological images from retrospective data were included in the internal validation set, which was used for hyperparameter tuning. The prospective data were designated as the external test set to assess the model’s generalization capability, with the number of samples in the external test set approximately matching that of the internal validation set in a 1:1 ratio. Additionally, the external test set included pathological image types that corresponded to those in the internal validation set, ensuring a consistent representation of lesion types across datasets.

All images were acquired using *in vivo* confocal microscopy (HRT III/RCM, Heidelberg Engineering, Germany). The study was conducted in accordance with the Declaration of Helsinki (as revised in 2013). The study was approved by the Ethics Committee of the People’s Hospital of Guangxi Zhuang Autonomous Region (No. KY-KJT-2021–70) and the requirement for informed consent was waived for this retrospective study.

#### 2.1.2 Data annotation and preprocessing

In this study, all images were subjected to binary classification to determine whether they belonged to the “anomalous” or “normal” category. Each image was independently evaluated by two board-certified ophthalmologists specializing in corneal diseases, each with over 5 years of clinical experience and expertise in interpreting corneal confocal microscopy images. Images with conflicting assessments or unclear evaluations from different specialists were excluded, while those with unanimous agreement among the specialists were assigned a definitive ground-truth classification label.

All identifying information was anonymized prior to analysis to ensure patient confidentiality. The original grayscale IVCM images were converted to RGB format by duplicating the single grayscale channel across the three RGB channels, as the model requires input in RGB format. All images were fed into the model at their original resolution of 384 × 384 pixels, after removing peripheral borders and overlaid text.

### 2.2 Transformer-based anomaly detection model

#### 2.2.1 Model structure

This study employs a transformer-based unified method for multi-class anomaly detection. On one hand, the model is trained using normal samples from multiple categories, enabling multi-level analysis of the cornea. On the other hand, it detects various types of diseases by identifying morphological deviations from normality. The anomaly detection is based on a reconstruction framework (Shi et al., 2021; You et al., 2022). For a query image, the model performs reconstruction during the prediction process and calculates the reconstruction error by comparing the original image with its reconstructed counterpart. Theoretically, a reconstruction model trained on normal images should succeed in reconstructing normal images but fail in abnormal images. Therefore, if the reconstruction error exceeds a predefined threshold, the original image is considered anomalous.

**FIGURE 1:**
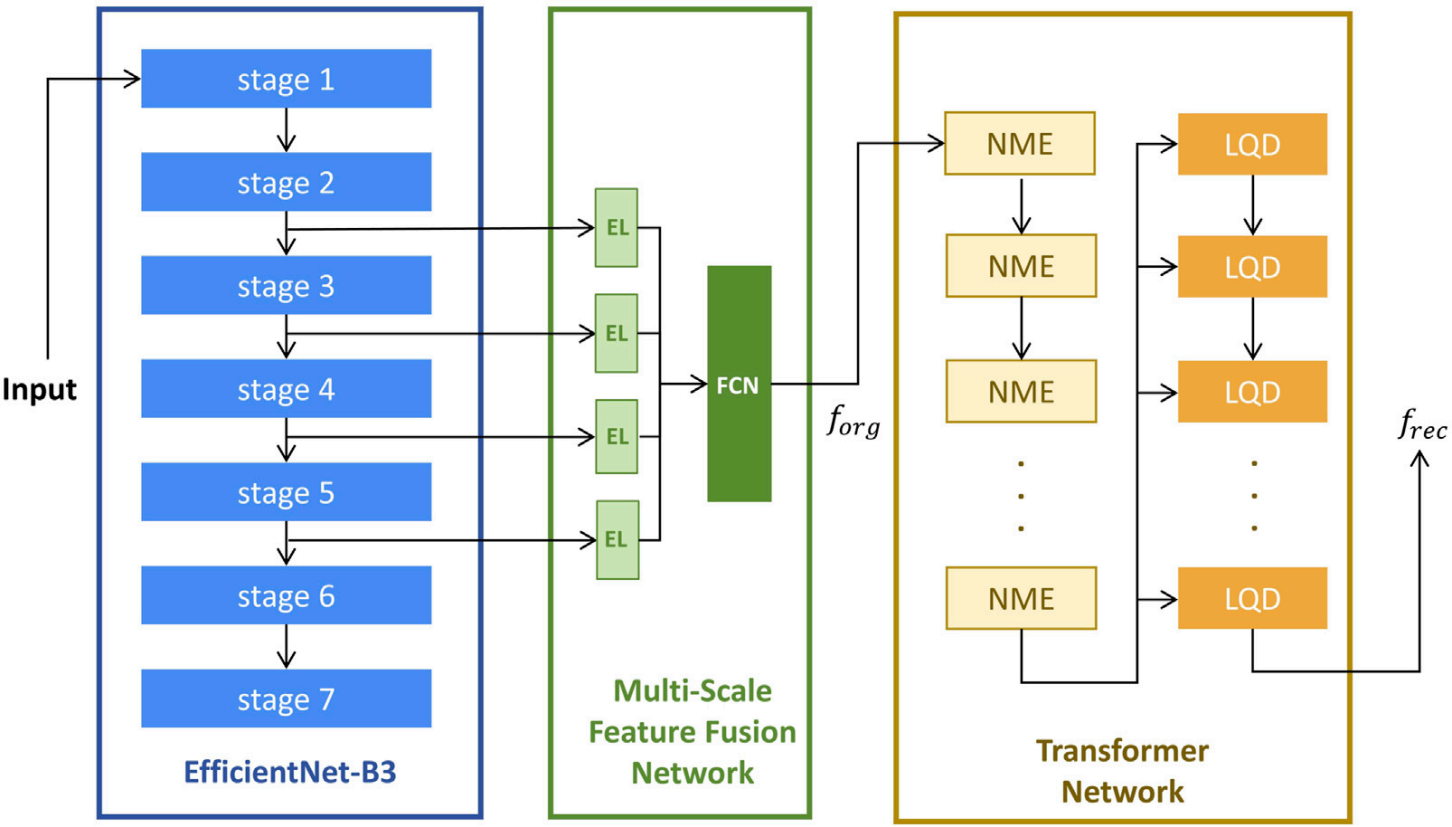
The Architecture of anomaly detection model. El, Embedding Layers; FCN, Fully Connected Network; NME, Neighbor Masked Encoder; LQD, Layer-wise Query Decoders. $f_{org}$ is a concatenated feature map formed by merging the extracted features along the channel dimension; $f_{rec}$ is the reconstructed image feature obtained from the final output of the last LQD.

The model architecture, as illustrated in Figure 1, consists of three sequential modules: a fixed EfficientNet-B3 network (Tan and Le, 2019) pre-trained on ImageNet (Deng et al., 2009), a Multi-Scale Feature Fusion Network (MFFN), and a Transformer network (Vaswani et al., 2017).

The Multi-Scale Feature Fusion Network (MFFN) consists of four embedding layers and a fully connected subnetwork designed to integrate multi-scale feature representations. Four input features are processed through separate Embedding Layers (EL) to transform them into a common-dimensional space. And then fed into a Fully Connected Network (FCN) comprising three fully connected layers, enabling hierarchical feature fusion and enhancing the model’s ability to capture complex patterns in original input images.

The Transformer network consists of multiple Neighbor Masked Encoders (NME) and Layer-wise Query Decoders (LQD) (You et al., 2022). In this architecture, both NME and LQD are repeated multiple times, and their interactions play a crucial role in refining the feature representations throughout the reconstruction process. The NME follows the standard architecture of a basic Transformer encoder, where each layer consists of an attention module and a Feed-Forward Network (FFN). In place of the traditional attention mechanism, the Neighborhood Masked Attention (NMA) is employed to prevent information leakage between neighboring regions. This modification enhances the model’s ability to focus on relevant features while maintaining spatial integrity during feature encoding. To improve the model’s expressive power, we use the LQD to strengthen the use of query embeddings. In addition, the learnable position embeddings (Kenton and Toutanova, 2019; Dosovitskiy et al., 2021) are added in attention modules to inform the spatial information.

The computation flow begins with the EfficientNet-B3 network, which is used for feature extraction from the input image. Features are selected from stage-2 to stage-5, which capture a range of spatial and semantic information at various scales. These extracted features are then passed into the MFFN, where they are fused into a unified feature map, denoted as $f_{org}$ . Subsequently, the feature map $f_{org}$ is fed into the Transformer network. The NME processes this input to generate encoder embeddings, which encapsulate the spatial and contextual relationships of the image features. In each LQD, the encoder embeddings are used as inputs along with the outputs from the previous LQD layer (self-fusion for the first LQD layer). This process iteratively refines the feature representations, and the final output of the last LQD is treated as the reconstructed image feature, denoted as $f_{rec}$ . The reconstruction error between $f_{org}$ and $f_{rec}$ is then computed to assess the anomaly of the input image.

#### 2.2.2 Training and prediction

During training, only normal samples are used, and the objective function is defined as the Mean Squared Error (MSE) loss between the original feature map $f_{org}$ and the reconstructed feature map $f_{rec}$ . The loss is calculated as:

$$Loss=\frac{1}{H\times W}{\left\|f_{org}-f_{rec}\right\|}_{2}^{2}$$

where H and W are the dimensions of the feature map.

For prediction, the model processes new images by computing the L2 norm of the pixel-wise differences between $f_{org}$ and $f_{rec}$ , then averaging these values across all pixels to obtain the reconstruction error for the image:

$$S=avg\left({\left\|f_{org}-f_{rec}\right\|}_{2}\right)$$

A image is classified as anomalous when the reconstruction error S for the given image exceeds a predefined threshold.

The key hyperparameters of the proposed method are summarized in Table 1.

**TABLE 1:** The hyperparameters of Proposed Method.

Efficientnet_B3	
Pretrained	TRUE
MFCN^†^	
Outstrides	16
Transformer	
Number of hidden dimension	768
Number of attention head	8
Number of encoder layer	7
Number of decoder layer	7
Dropout	0.1
Activation	relu
Optimizer	AdamW
Learning rate	0.0001
Weight decay	0.001
Batch size	8
Criterion	FeatureMSELoss
Size of neighbor mask	[9,9]

MFCN, Multi-Scale Feature Concatenation N.

### 2.3 Baseline algorithms

The proposed method is compared with several state-of-the-art baseline algorithms, including AnoGAN (Schlegl et al., 2017), G2D (Pourreza et al., 2021), and DRAEM (Zavrtanik et al., 2021), all of which follow a reconstruction-based anomaly detection approach.

AnoGAN consists of two main components: a generator and a discriminator. The generator is a convolutional decoder that maps random noise to normal images, utilizing a series of convolutional layers, leaky rectified linear units (LeakyReLU) activations, and batch normalization. The discriminator, which is a convolutional neural network, distinguishes between real images from the training set and the synthetic images generated by the generator. The model is trained adversarially, where the generator tries to create images that resemble normal samples, while the discriminator learns to differentiate between real and generated images. The key advantage of AnoGAN is its ability to successfully reconstruct normal images, with the reconstruction error increasing when applied to anomalous images, thus enabling anomaly detection.

G2D focuses on detecting irregularities by modeling the diversity of anomalies. It proposes that irregular samples can be viewed as deviations from regular instances in the training data. G2D introduces three main modules: the Irregularity Generator Network (I), Critic Network, and Detector Network (D). The irregularity generator (I) is trained on normal samples and is used to generate abnormal samples by introducing random deviations from the normal data. These generated anomalies are then used to optimize the parameters of the detector network (D), which distinguishes between normal and anomalous samples using a binary classification approach.

DRAEM integrates reconstructive and discriminative sub-networks for anomaly detection. The reconstructive sub-network focuses on detecting and reconstructing anomalies with semantically plausible content, while preserving non-anomalous regions. Simultaneously, the discriminative sub-network learns a joint reconstruction-anomaly embedding, which produces anomaly segmentation maps. DRAEM generates anomalous training examples by simulating anomalies on anomaly-free images, simulating diverse anomalous samples with pixel-perfect segmentation maps, eliminating the need for real anomalous samples.

All models were developed in Python programming language (Python Language Reference, version 3.7, Python Software Foundation) by PyCharm software (PyCharm Community Edition 2020.3.1, JetBrains). The training and testing were performed on an NVIDIA Tesla T4 GPU.

### 2.4 Data analysis

#### 2.4.1 Performance measurement

The performance of all four models was evaluated by plotting receiver operating characteristic (ROC) curves, which illustrate the relationship between the false positive rate (1-specificity) and the true positive rate (sensitivity). The areas under the ROC curves (AUCs) were calculated to assess the discriminatory power of each model for both internal and external validation datasets. The AUCs were statistically tested against the chance level (AUC = 0.5), with significance defined as p-value <0.05. The 95% confidence intervals (CIs) of the AUC values were reported for robust evaluation. Pair-wise comparisons of ROC between the models were made by MedCalc software according to the method proposed by DeLong et al. (1988).

Further performance metrics, including accuracy, sensitivity (recall), specificity, precision, and the Youden Index, were calculated for all models based on their respective confusion matrices. In this study, the optimal thresholds for each model were determined by identifying the point at which the Youden Index was maximized, thereby achieving a balance between sensitivity and specificity.

#### 2.4.2 Anomaly score analysis

The anomaly score generated by the model quantifies the extent to which a test sample deviates from the normal class. For the external validation dataset, the mean anomaly scores for the anomalous class (positive class) and the normal class (negative class) were calculated, and the 95% CI of the anomaly scores were reported. A Mann-Whitney U test was conducted to compare the anomaly scores between these two classes, given that the scores did not follow a normal distribution. Statistical analyses were conducted using SPSS (version 27.0, IBM Corp., Armonk, NY, USA), with a p-value <0.05 considered indicative of statistical significance.

The raw anomaly scores ranged from 0 to 255. For more intuitive interpretation, these scores were normalized to a range of [0,1] using the following formula:

$$Normalized\;score=\frac{S_{\max}-S}{S_{\max}-S_{\min}}$$

Where S represents the raw anomaly score of one image, $S_{\max}$ and $S_{\min}$ denote the maximum and minimum value of the raw anomaly scores in the internal validation set, respectively. In this study $S_{\max}$ is 70 and $S_{\min}$ is 5. To visualize the distribution of normalized anomaly scores, a grouped scatter plot was created for the external test dataset.

#### 2.4.3 Explainable anomaly map

To enhance the interpretability of the anomaly detection model, we visualize the reconstruction error to highlight regions identified as anomalous. Specifically, a difference feature map $f_{dif}$ representing pixel-wise reconstruction errors is first scaled to match the dimensions of the original input image. Next, a heatmap based on $f_{dif}$ is generated using a color gradient, where areas with higher reconstruction errors are displayed in red, indicating potential anomalies, and areas with lower errors are shown in blue, representing normal regions. Finally, the heatmap is overlaid onto the original input image, providing an intuitive visualization of the specific regions where anomalies are detected. This approach offers an accessible way to identify abnormal areas within the image.

## 3 Result

### 3.1 Clinical characteristics

**FIGURE 2:**
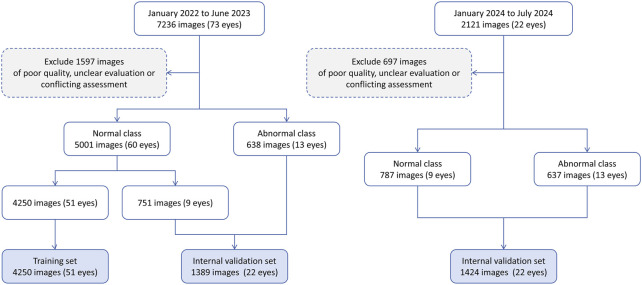
The workflow of this study.

A total of 9357 IVCM images were initially collected, comprising 7,049 images from healthy eyes and 2,308 images from pathological eyes. After excluding 2,294 images, 7,063 images were included in the study, consisting of 5,788 normal images from 69 healthy eyes and 1,275 anomalous images from 26 pathological eyes. Figure 2 shows the workflow for dataset development. The included pathological eyes represented the following conditions: infectious keratitis (fungal keratitis: four eyes, bacterial keratitis: four eyes, viral keratitis: four eyes, acanthamoeba keratitis: two eyes), non-infectious keratitis (filamentary keratitis: two eyes), corneal dystrophy (Fuchs endothelial dystrophy: two eyes), corneal degeneration (keratoconus: two eyes), corneal chemical burns (alkali burn: two eyes, acid burn: two eyes), and corneal leucoma (2 eyes). The training set included 4,250 normal images from 51 healthy eyes, the internal validation set included 751 normal images from nine healthy eyes and 638 anomalous images from 13 pathological eyes, and the external test set included 787 normal images from nine healthy eyes and 637 anomalous images from 13 pathological eyes. The characteristics of the samples are summarised in Table 2. No significant differences were found in age and gender among the training, internal validation and external testing sets (both p > 0.05).

**TABLE 2:** Characteristics of included samples.

Dataset Characteristics	Training set	Internal validation set	External test set
n of image (n of eye)	4,250 (51)	1,389 (22)	1,424 (22)
Category, n of image (n of eye)			
Normal	4,250 (51)	751 (9)	787 (9)
Abnormal	—	638 (13)	637 (13)
Fungal keratitis	—	123 (2)	128 (2)
Bacterial keratitis	—	118 (2)	126 (2)
Viral keratitis	—	130 (2)	119 (2)
Acanthamoeba keratitis	—	51 (1)	44 (1)
Filamentary keratitis	—	49 (1)	45 (1)
Fuchs endothelial dystrophy	—	31 (1)	27 (1)
Keratoconus	—	33 (1)	40 (1)
Alkali burn	—	41 (1)	34 (1)
Acid burn	—	36 (1)	44 (1)
Corneal leucoma	—	26 (1)	30 (1)
Patient characteristics			
Age, median (IQR)	31.0 (12.5)	43.5 (16.5)	46.5 (18.5)
Gender, n (%)			
Male	27 (52.9)	12 (54.5)	10 (45.5)
Female	24 (47.1)	10 (45.5)	12 (54.5)

IQR, interquartile range.

### 3.2 Anomaly detection performance

**FIGURE 3:**
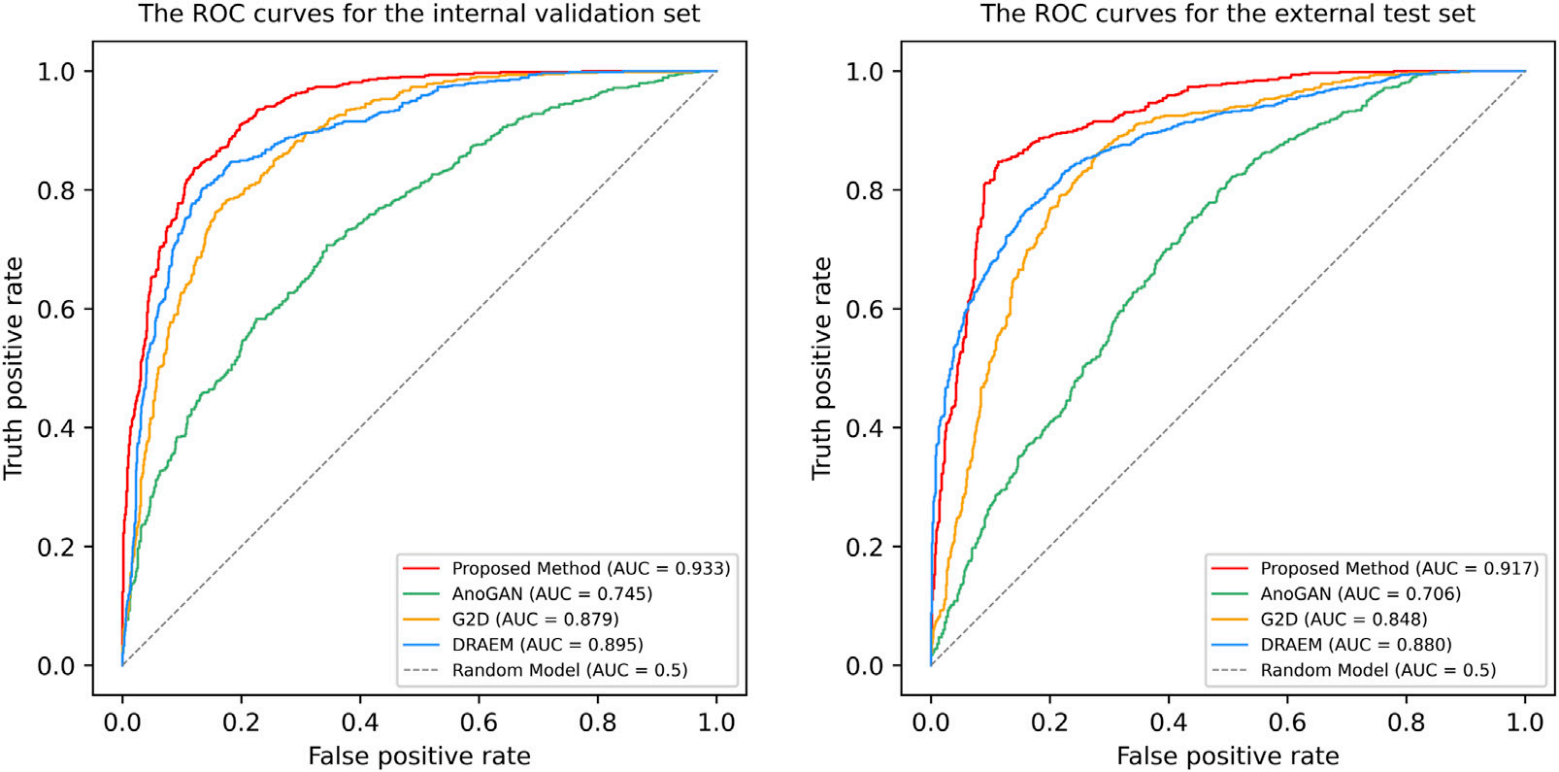
The Receiver operating characteristic (ROC) curves of four anomaly detection models in internal validation and external test. ROC, receiver operating characteristic curve; AUC, the areas under the ROC curve; AnoGAN, generative adversarial networks; G2D, generate to detect anomaly model; DRAEM, discriminatively trained reconstruction anomaly embedding model.

The ROC curves and AUC values for the proposed Transformer-based model, DRAEM, G2D, and AnoGAN are shown in Figure 3. The proposed model achieved the highest AUC values, with 0.933 (95% CI: 0.921–0.946, p < 0.001) for internal validation and 0.917 (95% CI: 0.902–0.931, p < 0.001) for external testing, demonstrating its superior discriminatory performance compared to the baseline models. In both validation stages, DRAEM followed as the second-best model, with G2D and AnoGAN showing progressively lower AUC values. The 95% confidence intervals (CIs) of the AUC values for the proposed model did not overlap with those of the baseline models. Furthermore, DeLong’s test revealed that all pairwise comparisons between the proposed model and each baseline model yielded p-values <0.001, indicating statistically significant differences in performance. These results underscore the effectiveness of the proposed method in distinguishing between normal and anomalous images, outperforming all other models in terms of discriminatory power.

**FIGURE 4:**
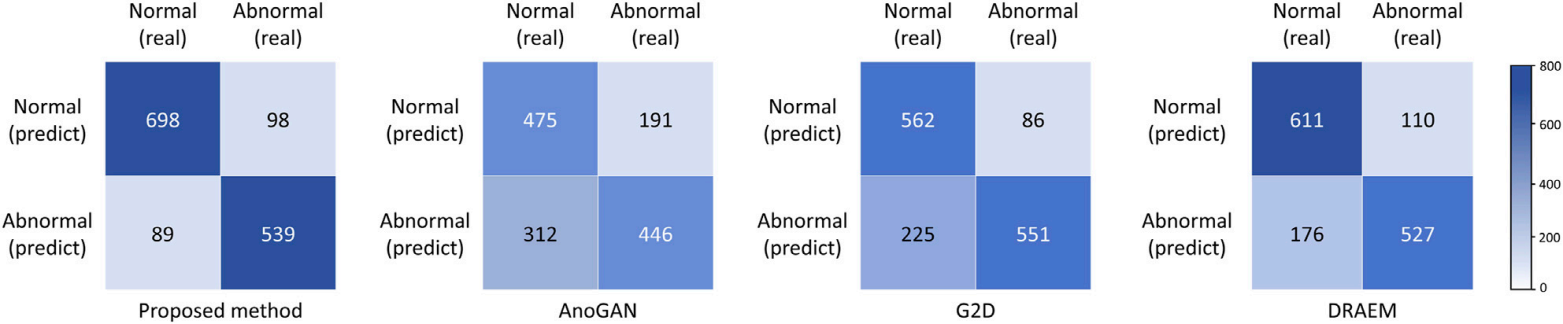
Confusion matrices of four anomaly detection models in external test. AnoGAN, generative adversarial networks; G2D, generate to detect anomaly model; DRAEM, discriminatively trained reconstruction anomaly embedding model.

Performance metrics, including accuracy, sensitivity, specificity, precision, and the Youden Index, are summarized in Table 3, with confusion matrices displayed in Figure 4. The proposed method outperformed all baseline models across all metrics, achieving an accuracy of 0.869, a sensitivity (recall) of 0.846, a specificity of 0.887, a precision of 0.858, and Youden Index value of 0.733. DRAEM ranked second, followed by G2D and AnoGAN, consistent with the trends observed in the AUC results.

**TABLE 3:** Performance of anomaly detection models in external validation.

Model	AUC (95% CI)	Accuracy	Sensitivity (recall)	Specificity	Precision	Youden Index
Proposed method	0.917 (0.902–0.931)	0.869	0.846	0.887	0.858	0.733
AnoGAN	0.706 (0.679–0.732)	0.647	0.700	0.604	0.588	0.304
G2D	0.848 (0.828–0.868)	0.782	0.865	0.714	0.710	0.579
DRAEM	0.880 (0.862–0.898)	0.799	0.827	0.776	0.750	0.604

AUC, The area under the ROC curve; CI, confidence intervals; AnoGAN:generative adversarial networks; G2D, generate to detect anomaly model; DRAEM, discriminatively trained reconstruction anomaly embedding model.

### 3.3 Anomaly score analysis

For our proposed model, the mean anomaly score for the anomalous class (positive class) in the external test dataset was 24.53 (95% CI: 24.27–24.80), while the mean score for the normal class (negative class) was 20.08 (95% CI: 19.95–20.22). The difference in mean anomaly scores between the two classes was statistically significant (p < 0.001), indicating that our proposed model effectively distinguished between normal and pathological images based on anomaly scores.

**FIGURE 5:**
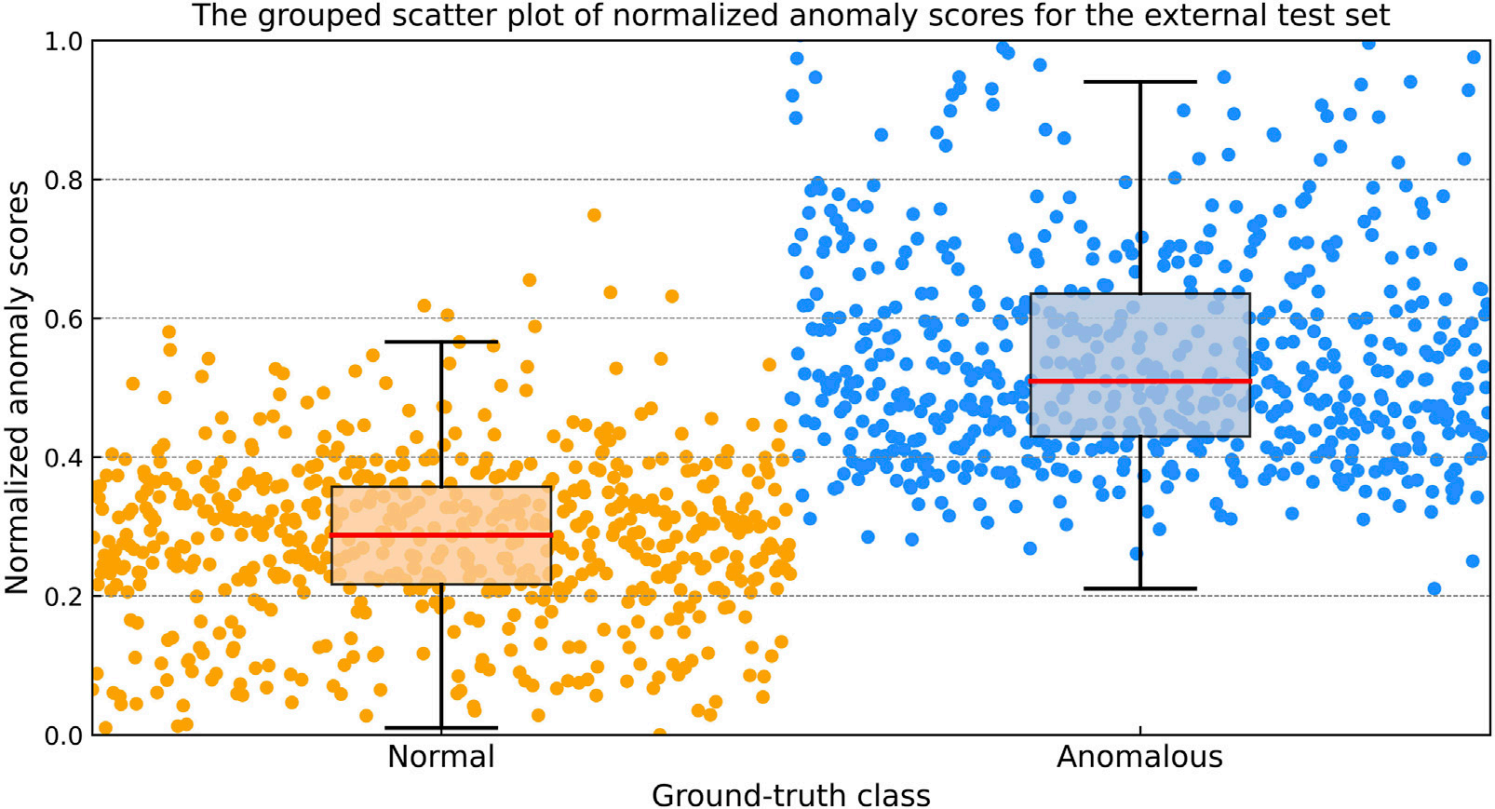
The grouped scatter plot of normalized anomaly scores for the external test dataset. Samples from the anomalous class exhibited higher normalized anomaly scores compared to those from the normal class, with minimal overlap between the two groups.

The grouped scatter plot (Figure 5) illustrates the distribution of normalized anomaly scores for the external test dataset. Samples from the anomalous class exhibited higher normalized anomaly scores compared to those from the normal class, with minimal overlap between the two groups.

### 3.4 Explainable anomaly map

**FIGURE 6:**
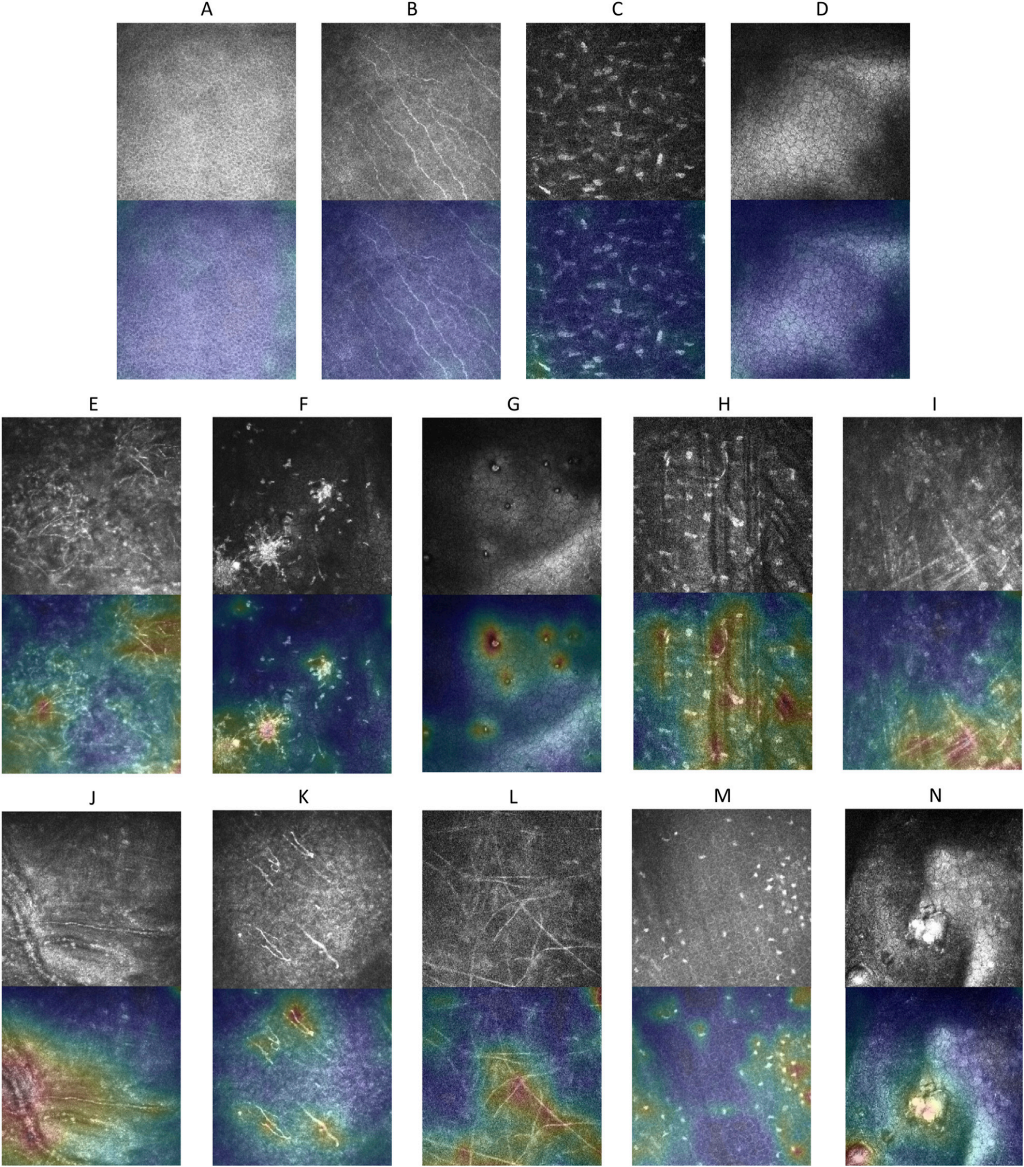
Explainable anomaly maps for normal and abnormal corneal images. **(A–D)** Show the original images and their corresponding explainable anomaly maps for healthy corneal layers, including the epithelium, subepithelial nerve plexus, stroma, and endothelium. **(E–N)** Display the original images and explainable anomaly maps for various types of pathological corneal abnormalities.

Explainable anomaly maps were generated for our proposed model on the external test dataset to visualize the regions identified as anomalous (Figure 6). The maps effectively highlighted areas with structural deviations in images from diseased corneas, while showing minimal activation in healthy samples. Across various pathological conditions, the anomaly maps consistently outlined regions corresponding to morphological abnormalities, demonstrating our proposed model’s ability to localize potential anomalies without prior knowledge of specific disease types.

## 4 Discussion

In this study, we developed a Transformer-based unsupervised anomaly detection method for IVCM images, achieving an AUC of 0.933 during internal validation and 0.917 on an external test dataset. Our model outperformed existing methods, such as AnoGAN, G2D, and DRAEM, in terms of both performance and generalizability. It effectively distinguished normal corneal images from pathological ones, with statistically significant differences in anomaly scores between the two classes. Additionally, we incorporated visual interpretability to enhance understanding of the model’s decision-making process, further demonstrating its robustness.

A key innovation of this study is the development of a unified anomaly detection model capable of addressing the challenges posed by diverse disease manifestations. As an initial diagnostic step, our model efficiently identifies images with potential lesions from large datasets, allowing ophthalmologists to prioritize high-risk patients. Unlike traditional methods, it can identify a wide range of diseases—including previously unseen types during training—without requiring prior knowledge of their features, thereby improving the efficiency of automated image analysis while reducing computational overhead. This unified approach reduces the need for separate models for each disease, mitigating the risk of missed diagnoses and providing a scalable solution for clinical applications.

Another distinctive feature of our study is the ability to train a unified model to handle the morphological diversity within normal samples. While transformer-based techniques have been explored for anomaly detection in other imaging modalities such as OCT and retinal images, our study tackles a significantly different challenge. In OCT and retinal images, the normal class typically has a relatively uniform morphology. However, in the case of IVCM images, the normal class encompasses multiple subcategories with substantial morphological variations. This complexity necessitates that the model learns to discern the distinct features of these diverse normal subcategories, thereby enabling it to effectively distinguish abnormalities from the entire spectrum of normal variations, rather than being confounded by the intra-class variability among the normal subcategories.

Specifically, the cornea consists of the epithelium, Bowman’s layer, stroma, Descemet’s membrane, and endothelium. Bowman’s layer, characterized by the presence of a nerve plexus, was categorized as the sub-basal nerve plexus layer in this study. Descemet’s membrane, due to its scarcity and frequent overlap with stromal or endothelial features, was integrated into these categories to ensure robust learning. To accommodate this diversity, we subdivided normal IVCM images into four subclasses, enabling the model to learn and distinguish multiple normal features simultaneously, thus overcoming the challenge of multi-class normality representation.

As demonstrated in the results, the AnoGAN algorithm exhibits the poorest performance, primarily due to its reliance on a relatively shallow network architecture with only a few convolutional layers. This architecture, while effective for simpler images, struggles to extract complex features from IVCM images. The AUC values of G2D and DRAEM are lower than those of the proposed method, mainly because these models are designed to reconstruct normal images with consistent features. However, IVCM images consist of multiple distinct layers, which these models struggle to handle. As a result, they tend to overfit to a specific layer (typically the layer with the most data in the training set), which limits their ability to generalize.

In contrast, the superior performance of the proposed method can be attributed to its three-stage architecture, which not only captures the multi-scale features of IVCM images but also uses the transformer architecture for reconstruction. The fixed EfficientNet-B3 network, pre-trained on ImageNet, serves as a powerful feature extractor, leveraging its deep hierarchical structure to capture both fine-grained and high-level representations. Building upon these extracted features, the Multi-Scale Feature Fusion Network (MFFN) aligns and integrates multi-scale representations through separate embedding layers and a fully connected network, ensuring that diverse structural details from different spatial scales are effectively combined. Furthermore, the Transformer network, equipped with Neighbor Masked Encoders (NME) and Layer-wise Query Decoders (LQD), progressively reconstructs the image by refining feature representations at each stage. The use of Neighborhood Masked Attention (NMA) is designed to prevent the encoders and decoders from directly reconstructing the image, thereby improving the model’s generalization ability and avoiding overfitting to a specific layer of the IVCM image. This sequential feature extraction, fusion, and reconstruction process allows the model to fully exploit the structural complexity of IVCM images, leading to improved anomaly detection performance.

Training exclusively on normal samples offers significant practical advantages. By eliminating the need for abnormal samples, this approach significantly simplifies data preparation and circumvents the challenges associated with obtaining sufficient annotated data for diseases. In real-world clinical scenarios, disease samples are often scarce due to limited availability and high annotation costs, particularly for rare conditions. Training exclusively on normal samples not only addresses these limitations but also ensures the model’s applicability to a broad range of pathologies.

In this study, explainable anomaly maps were utilized to visualize the regions identified as anomalous by the model, providing critical insights into its decision-making process. By highlighting areas of structural deviation in pathological images, these maps often correspond to morphological abnormalities, suggesting their potential as imaging biomarkers for corneal diseases. This localized visualization provides a foundation for understanding disease-specific features while improving the model’s transparency and clinical applicability.

The limitations of this study warrant consideration. First, the model was developed and validated using single-center data, which may limit its ability to capture the variability in imaging conditions and patient populations encountered in broader clinical settings. Future studies incorporating multi-center data are warranted to improve its generalizability. Second, while the model effectively identifies anomalies, it does not classify specific abnormal findings. This is consistent with its intended role as an initial screening tool in the diagnostic workflow. By focusing on detecting deviations from normal structures, the model enables rapid identification of potential abnormalities, streamlining subsequent evaluations by clinicians or specialized diagnostic models. Third, by analyzing the misclassification cases, we found that false positives often involved normal images with uncommon features such as thick stromal nerve fibers or transitional zones between corneal layers with atypical morphology. These clinically normal variations may fall outside the learned distribution due to limited representation in the training set. False negatives were typically early-stage anomalies with subtle deviations resembling normal patterns, highlighting the model’s limited sensitivity to minor pathological changes. Future improvements will focus on expanding the diversity and heterogeneity of normal training data to better capture the full range of physiological variation. Finally, the visualization approach identifies anomalies based on morphological deviations from normal structures; however, these deviations may not always correspond to clinically significant findings. Further research is needed to establish the clinical relevance of the detected anomalies and enhance the model’s ability to prioritize diagnostically meaningful abnormalities.

## 5 Conclusion

This study establishes a robust and interpretable unsupervised anomaly detection method for IVCM images, effectively addressing the complexity of corneal morphology. By training exclusively on normal images, the model demonstrated high accuracy in detecting various anomalies and reliably distinguishing pathological images from normal ones. Its unified design enhances anomaly screening, significantly improving diagnostic efficiency and offering broad potential for clinical applications.

## Data Availability

The raw data supporting the conclusions of this article will be made available by the authors, without undue reservation.
